# Bioinformatics of germline variant discovery for rare disease diagnostics: current approaches and remaining challenges

**DOI:** 10.1093/bib/bbad508

**Published:** 2024-01-23

**Authors:** Yury A Barbitoff, Mikhail O Ushakov, Tatyana E Lazareva, Yulia A Nasykhova, Andrey S Glotov, Alexander V Predeus

**Affiliations:** Dpt. of Genomic Medicine, D.O. Ott Research Institute of Obstetrics, Gynaecology, and Reproductology, Mendeleevskaya line 3, 199034, St. Petersburg, Russia; Bioinformatics Institute, Kentemirovskaya st. 2A, 197342, St. Petersburg, Russia; Dpt. of Genomic Medicine, D.O. Ott Research Institute of Obstetrics, Gynaecology, and Reproductology, Mendeleevskaya line 3, 199034, St. Petersburg, Russia; Dpt. of Genomic Medicine, D.O. Ott Research Institute of Obstetrics, Gynaecology, and Reproductology, Mendeleevskaya line 3, 199034, St. Petersburg, Russia; Dpt. of Genomic Medicine, D.O. Ott Research Institute of Obstetrics, Gynaecology, and Reproductology, Mendeleevskaya line 3, 199034, St. Petersburg, Russia; Dpt. of Genomic Medicine, D.O. Ott Research Institute of Obstetrics, Gynaecology, and Reproductology, Mendeleevskaya line 3, 199034, St. Petersburg, Russia; Bioinformatics Institute, Kentemirovskaya st. 2A, 197342, St. Petersburg, Russia

**Keywords:** NGS, WES, variant calling, variant annotation, reference genome

## Abstract

Next-generation sequencing (NGS) has revolutionized the field of rare disease diagnostics. Whole exome and whole genome sequencing are now routinely used for diagnostic purposes; however, the overall diagnosis rate remains lower than expected. In this work, we review current approaches used for calling and interpretation of germline genetic variants in the human genome, and discuss the most important challenges that persist in the bioinformatic analysis of NGS data in medical genetics. We describe and attempt to quantitatively assess the remaining problems, such as the quality of the reference genome sequence, reproducible coverage biases, or variant calling accuracy in complex regions of the genome. We also discuss the prospects of switching to the complete human genome assembly or the human pan-genome and important caveats associated with such a switch. We touch on arguably the hardest problem of NGS data analysis for medical genomics, namely, the annotation of genetic variants and their subsequent interpretation. We highlight the most challenging aspects of annotation and prioritization of both coding and non-coding variants. Finally, we demonstrate the persistent prevalence of pathogenic variants in the coding genome, and outline research directions that may enhance the efficiency of NGS-based disease diagnostics.

## INTRODUCTION

The emergence of next-generation sequencing (NGS) technology has transformed genetics and genomics. In contrast to earlier methods, nucleotide sequences of millions and billions of DNA fragments can be determined in a single NGS experiment. Rapid development and spread of NGS led to a dramatic reduction of sequencing costs, and made genomic analysis available for both researchers and clinicians. The most commonly used NGS platforms (such as Illumina) are based on short (usually 100–150 bp) sequencing reads (reviewed in [1]), though novel methods based on longer reads are becoming more popular (reviewed in [2]).

In medical genetics, NGS is applied to search for the alterations of the genome sequence (genetic variants) that cause rare inherited diseases [3]. These include both relatively simple variants, such as single-nucleotide variants (SNVs) or short (up to several dozen base pairs) insertions or deletions (indels), and more complex types of variants, such as complex chromosomal rearrangements (structural variants, SVs) or copy number variants (CNVs). Although the general goal of the analysis is relatively straightforward, many caveats remain which hinder efficient discovery of such causal genetic variants. It has been reported that the diagnosis rate from whole-genome sequencing (WGS) in trios is limited to a mean of 42%, and this rate significantly varies between different disease groups (reviewed in [4]). The diagnosis rate may also decrease if approaches other than WGS are used, such as whole-exome sequencing (WES) (i.e. targeted sequencing of all protein-coding sequences of the genome) or sequencing of target gene panels.

Bioinformatic analysis and interpretation is the core step in NGS-based diagnostics of rare disease. The main goal of bioinformatic analysis of NGS data in medical genetics is the comprehensive identification of all genetic variants present in the sample. Interpretation of NGS results, on the other hand, concerns with further filtering and classification of the identified variants with a goal of finding one or several variants causally linked to the patient’s phenotype. The efficiency of molecular diagnostics with NGS methods depends on multiple factors, and the incompleteness of knowledge regarding the pathogenetic mechanisms behind rare disease is arguably the most important of such factors. At the same time, data analysis and interpretation also represent a major challenge, and certain persistent problems and user errors may negatively impact the accuracy of the analysis and the diagnosis rate.

**Figure 1:**
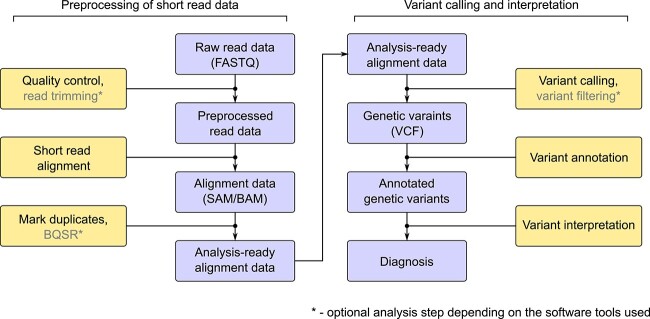
Basic workflow of NGS data analysis in rare disease diagnostics. The diagram shows the principal steps of the analysis pipeline.

The main data analysis steps that are necessary for causal genetic variant identification are summarized in Figure 1. First, raw sequencing reads in the FASTQ format [5] are subjected to quality control (QC) and preprocessing (though the latter step may be omitted in some workflows, see next). After initial QC, reads are aligned to the reference genome sequence, and the alignment results in SAM or BAM format [6] are subjected to additional processing and refinement steps. The processed alignment data are used for variant calling, resulting in a raw VCF file containing all detected variants. This file may undergo additional filtering (depending on the variant calling software used), and then annotated using a variety of available software and resources. Annotated VCF file may then be used for variant interpretation, leading to selection of a variant (or variants) that are considered pathogenic (or likely pathogenic) and potentially causal for the disease in question. Presence of this variant is typically validated by visual inspection of alignments and Sanger sequencing [7].

In this review, we will discuss in more detail current bioinformatic approaches and remaining challenges in each of the aforementioned steps. Despite the ongoing introduction of long reads to medical genetics and their undoubtful advantages for SV analysis, we will focus on the analysis of short read data, which are most commonly used in rare disease diagnostics.

## BASIC DATA ANALYSIS WORKFLOW FOR GERMLINE VARIANT CALLING

There exist a plethora of methods for performing the main analysis steps; hence, we did not intend to provide a comprehensive list of all software. Instead, next we will briefly outline the main analysis steps and highlight the best-performing methods based on recent benchmarks.

### From raw data to analysis-ready alignments

Bioinformatic analysis of data starts with the raw sequencing reads. The reads are usually stored in a FASTQ file [5]. QC of raw sequencing reads is performed to examine the base qualities, nucleotide composition and adapter content of the reads. This step is almost exclusively performed using the FASTQC tool or multiQC [8], and may provide insights into the general quality of the sequencing run and library preparation. However, raw reads QC is not enough to conclude that the sequencing data are of sufficient quality, as many important features of the data cannot be examined without read alignment or variant calling results (see next).

After the initial QC step, additional preprocessing of reads may be performed to eliminate adapter sequences using tools such as FASTP [9] or Trimmomatic [10]. This step, however, may be omitted if the results of raw data QC do not indicate presence of detectable adapter sequences. Furthermore, trimming of adapters has been shown to have minimal effects on variant calling in both bacteria [11] and humans [12] in real-life datasets.

Alignment of reads onto the reference genome is typically performed using the BWA MEM [13, 14] tool. However, other well-performing options exist for read alignment, such as Isaac4 (https://github.com/Illumina/Isaac4) or Novoalign (http://novocraft.com/novoalign/). Our analysis of aligner performance showed that all commonly used tools perform similarly [15], with the exception of Bowtie2 [16], which should not be used for germline variant analysis. A more recent study showed that the Bowtie2 alignments can be used for variant calling, if the algorithm parameters are finely tuned, and the mapping quality scores are adjusted [17]. This finding, however, still argues against the routine usage of Bowtie2. Irrespective of the aligner, alignment data are stored in SAM or BAM format, and the SAMtools [6] is the standard tool to perform such operations as format conversion, sorting and indexing for alignment formats.

Mapped reads usually undergo another set of preprocessing steps. The first and the most common one is the identification of duplicate reads. Duplicate reads emerge for several reasons such as library amplification (PCR duplicates), as well as clustering or signal detection errors (optical duplicates). Duplicates introduce bias into the statistical assessment of candidate genetic variants, and marking of such reads helps to increase the accuracy of subsequent variant analysis. Marking of duplicate reads is commonly performed using the Genome Analysis ToolKit (GATK) [18] functionality; however, alternative solutions with better resource efficiency have been proposed, such as dopplemark (https://github.com/grailbio/doppelmark) or sambamba [19].

After marking duplicates, two additional alignment processing steps may be performed. The first of such additional steps is indel realignment, which is performed to correct alignment errors for reads bearing insertions or deletions near the start or end of the read. By default, such reads have a high probability of being soft-clipped by the alignment software or aligned with multiple mismatches instead of a gap. Indel realignment used to be a part of the standard GATK Best Practices workflow [20]; however, this procedure is now performed as part of the HaplotypeCaller algorithm. At the same time, other tools to solve the local realignment task have been developed, such as ABRA2 [21]. Another alignment processing step is the base quality score recalibration (BQSR), initially proposed by the GATK team [22]. The main goal of BQSR is to correct for the inaccuracies and biases in the base quality score estimates produced by the base calling software, which are known to be confounded by machine cycle and sequence context [22]. However, the effects of both indel realignment and BQSR on variant calling are subtle [23], and these steps can be omitted when using non-GATK variant calling in subsequent pipeline steps.

### Calling of short variants

Genetic variants can be broadly separated into two groups: (i) short variants, i.e. single-nucleotide substitutions or insertions/deletions up to 50 bp in length; and (ii) SVs that include longer and more complex rearrangements, such as large insertions, duplications or deletions, as well as inversions and translocations. In principle, both types of variants can be called in two different ways: in single-sample mode or in cohort mode (for a set of samples). Each analysis type, however, requires specifically designed software.

Short variant discovery is relatively simple and straightforward compared with SV analysis. There are two principal approaches to short variant calling. Pileup-based callers utilize a compressed representation of the alignment information for each position, and usually call variants based on the prevalence of non-reference bases in reads. While this idea is utilized by the simplest variant callers such as SAMtools [6] and VarScan [24], such an approach can be extremely powerful when combined with sophisticated deep learning-based algorithms (e.g. DeepVariant [25] and Clair3 [26], with the latter tool primarily designed for long-read sequencing data). The other approach to short variant discovery is haplotype-based calling which includes local assembly of reads into haplotypes, usually followed by re-alignment of reads and genotyping. Such an approach is implemented in GATK HaplotypeCaller [20, 22], which is the most widely used germline variant calling software. Other haplotype-based methods include Freebayes [27], Strelka2 [28] and Octopus [29]. Machine learning (ML) algorithms are also used in haplotype-based callers; however, in this case ML is used for subsequent variant filtering rather than the calling itself. For example, a random forest filtering model is available in Octopus, while GATK includes filtering options based on convolutional neural networks (CNNs) [30].

All of the aforementioned software tools are usually used for single-sample variant calling. In contrast to single-sample mode, a particular benefit of the joint calling approach is that all non-missing genotypes (including reference homozygous) are reported in a final VCF file for all samples in a cohort, which is especially important for statistical comparison of variant frequencies, association studies and family-based studies. Furthermore, joint calling was shown to increase the statistical power of variant calling [7, 22]. To perform cohort genotyping, one needs to obtain candidate variants (usually, stored in a GVCF format) for each sample, and then combine these files and jointly genotype the cohort. Several variant callers support GVCF output and can perform joint calling of several samples (e.g. Octopus, Strelka2); at the same time, only GATK natively supports joint genotyping for large cohorts. A recently proposed software tool called GLnexus [31] enables cohort genotyping for GATK- and DeepVariant-generated GVCFs.

Given that the number of tools for short variant calling is large and growing, regular evaluation of their performance is crucial for the proper choice of instruments. Such benchmarking of variant callers is usually performed using gold standard datasets. Several such datasets are provided by the Genome In A Bottle (GIAB) consortium [32]. These include publicly available WES and WGS data for members of the three trios of different ethnicity [Central European from Utah (CEU) individual (HG001, also known as NA12878), the Ashkenazi trio (HG002, HG003, HG004) and the Chinese trio (HG005, HG006, HG007)]. These samples are constantly resequenced using various sequencing technologies. For example, Baid *et al*. made an effort to extensively sequence all members of the three GIAB trios using both short and long reads [33]. Another option for benchmarking is to use the synthetic diploid sequence [34].

Recently, we made an effort to systematically benchmark these tools using a set of gold standard WES and WGS datasets from GIAB. Our analysis demonstrated that DeepVariant showed the best performance across all datasets and variant types [12, 15]. More importantly, neither DeepVariant nor any other ML-based caller showed evidence of being overfitted for GIAB data, and all methods performed similarly on gold standard data and unrelated samples of diverse ethnicities. At the same time, we demonstrated that ML-based methods other than DeepVariant may be sensitive to high coverage, in particular, in WES samples. Similarly, significant presence of adapters in reads may negatively affect pileup-based methods such as Clair3 [12].

Another important issue is filtering of called variants, which is commonly advised to reduce the burden of false positive calls. Such filtering, however, may be problematic in a diagnostic framework. For example, variant filtering models in GATK may yield puzzling results in high-coverage WES datasets [15]. Similarly, variant filtering methods for cohort genotyping (available in GATK or GLnexus) can also be quite prone to errors if data quality and/or cohort size are below expectation, and several comparisons reported no performance gain from variant filtering in cohorts (e.g. [25]). As thus, additional quality-based filtering of variants has to be approached with much caution. It may be advised to keep filtered variant calls accessible to the interpreter, so that they may be, if necessary, inspected during reanalysis.

It is also important to note that the benchmarking of variant calling pipelines is useful not only for comparison of different tools, but also for evaluation of the validation of pipeline prior to its use in clinical practice. However, such validation should not be limited to evaluation using gold-standard datasets, and specific guidelines for pipeline validation have been compiled recently (e.g. [35, 36]). Recent European recommendations for WGS suggest including monozygotic twins datasets as well as previously solved cases into optimization procedure to confirm the correct variant detection and annotation [37]. Additional evaluation of the pipeline is also advised when recent tool versions or additional functionality are implemented [37].

Furthermore, QC, including evaluation of the variant call quality (reviewed in [38]), has to be performed at all analysis stages routinely [37]. A particularly important problem in the data QC for diagnostic purposes is the detection of sample contamination and sample swaps. Detection of contamination in human DNA sequencing is more difficult than cross-species contamination, and bioinformatic methods for contamination analysis have to rely on patterns of sequence variation. Most of the methods utilize read alignment data to assess contamination. The most commonly used tool, VerifyBamID, uses population allele frequency information from external sources to estimate the contamination rate in a maximum likelihood framework [39]. Recently, an ancestry-agnostic version of the tool, VerifyBamID2, was released [40]. In addition to alignment-based solutions, several methods for contamination analysis have been developed that work with variant call data in VCF files. These include such tools as Peddy [41] or QC3 [42]. These tools provide useful metrics such as X chromosome variant counts, general heterozygous-to-homozygous variant ratio and estimated cross-sample relatedness. All of these metrics are useful both to identify sample swaps and significant levels of contamination. Recently, a new approach to contamination analysis, Contamination from Homozygous Alternate Reference Reads (CHARR), was proposed [43]. This method is based on the so-called ‘reference allele infiltration’ and may be used to rapidly detect different levels of contamination with comparable or better efficiency than VerifyBamID.

### SV and CNV calling

SV calling is markedly more complicated compared with short variant discovery, warranting a separate discussion. The detection of SVs is much more accurate with long read technologies, such as Oxford Nanopore (ONT) and Pacific Biosciences (PacBio). In particular, much higher sensitivity of SV detection is usually reported with long reads, and significantly more SVs can be identified in the same sample with long read sequencing (e.g. [44]). Nevertheless, short read data can also be used for this type of analysis, though with lower efficiency. Currently, there are multiple tools for SV detection from short reads, with DELLY [45], Manta [46] and Lumpy [47] being the most widely used ones (according to Google Scholar citations). Manta is also a part of the GATK-SV, an integrative multi-algorithm pipeline used to construct the largest publicly available SV dataset, gnomAD-SV [48]. New tools are actively being developed, such as Dysgu, which is also suitable for long reads [49], and ClinSV, which claims to achieve clinical-grade calling of SVs from short read data [50].

Different SV calling approaches have recently been reviewed by Mahmoud *et al*. [51]. Most of the commonly used software tools rely on read alignment results. Despite methodological differences, all of such tools utilize three types of information: paired read discordance, split reads and coverage depth. It is generally recommended to employ tools that integrate all of the aforementioned types of information in conjunction with a local read assembly [52]. GRIDSS [53] and Manta have been shown to be strong candidates, although the former reports candidate variants in the so-called breakend notation, which can pose challenges in terms of subsequent interpretation.

Regardless of their popularity, short-read-based methods appear to have hit a ceiling in terms of precision and recall. Frequently, these methods exhibit limitations in specific genomic regions such as low complexity, highly variable or highly repetitive regions [51]. It is noteworthy that the performance of SV calling tools can vary substantially depending on the size of the variant. As has been shown for deletions, nearly all methods tend to underestimate or overestimate the size of rearrangements [54]. Efforts have been made to address these challenges through ensemble calling, which involves combining the strengths of multiple tools to generate a consensus set of SVs (e.g. Parliament2 [55]); however, some studies suggest that using a single state-of-the-art tool is a more efficient approach [52].

One group of SVs that deserve a separate discussion are alterations in the number of copies of DNA segments termed copy number variations (CNVs). CNVs are mainly composed of deletions and duplications and constitute a significant portion of the human genome, accounting for approximately 4.8–9.5% of its total length (reviewed in [56]). To date, several dozen tools have been developed for the identification of CNV using short-read NGS data. The main limitation of these methods lies in detecting short variations that range in length from 50 to 500 bp [57]. Despite the broad range of algorithms available for CNV detection, the primary source of information utilized by the majority of them is read-depth (RD), which is also referred to as read-count (RC). RD-based methods can be classified into two main categories: reference-based methods, and those that detect CNV by comparing the RC of specific regions with the overall chromosome coverage (single sample-based methods). Most commonly used examples include CNVkit [58] and GATK gCNV [59]. Reference-based CNV detection requires a joint analysis of multiple samples, the presence of a pool of control samples or a corresponding model that has been trained on such a pool. Reference-based methods are generally advised for working with data types other than WGS, and accurate selection of reference samples is crucial to achieve higher accuracy [60].

Selection of an optimal CNV caller is complicated by low reproducibility of tool performance across different datasets, as well as limitations associated with the algorithm (e.g. reference-based versus single sample-based). More importantly, however, there is no gold standard dataset for a comprehensive benchmarking of CNV callers [61]. In recent years, several benchmark studies of various sizes have been conducted [61–65]. Making robust conclusions from these studies, however, has proven to be challenging due to the limited overlap in the methods they examine. Common observations from these studies include a biased distribution of detected CNV lengths among different tools and a generally low precision-recall ratio for both WES and WGS data analysis. Notably, the performance of methods for CNV detection from targeted gene panels was found to be significantly better [64], with DECoN [66] and panelcn.MOPS [67] exhibiting the highest metrics. Similarly to generic SV calling, new methods for CNV detection are frequently introduced (e.g. SawyCNV [68]), and ensemble-based approaches have demonstrated potential performance benefits, though no reliable solution using this approach has been provided yet [69].

### Calling mosaic variants from short-read data

Mosaic variants (variants present in a fraction of the cells of an organism that arise during development) are responsible for a substantial fraction of rare disease. Both short variants and SVs can be mosaic, and notable examples of both mosaic SVs and SNVs have been described in the context of congenital rare disorders, such as neurological or developmental (e.g. [70–72]).

While mosaic variants are somatic, calling of such variants is different from somatic mutations in cancer, where a normal tissue sample is usually used to filter candidate variants. In the absence of the control sample, separation of bona fide mosaic variants from inherited germline variants, contamination or technical noise becomes a crucial and complicated task. Specialized variant calling tools have been developed to detect mosaic variants with the help of linked reads technology (e.g. Samovar [73] or LinkedSV [74]). Recently, Wang *et al*. have proposed a set of best practices for identifying mosaic variants from short-read WGS data [75]. The proposed method includes identification of candidate mosaic variants followed by extensive filtering to exclude germline variants (based on variant allele frequency and frequency in the population), sequencing errors and variants outside of accessible genomic regions (see next). According to these best practices, the initial discovery of candidate variants can be performed using either somatic (MuTect2 [76]) or germline (GATK HaplotypeCaller with the ploidy parameter of 50) callers. Still, all methods tend to reach a sensitivity limit around 65%, despite deep ( 250x) WGS data being used for the analysis [75].

## KEY FACTORS AFFECTING THE ACCURACY OF VARIANT DISCOVERY

### The reference genome quality

Alignment is the first major step of the NGS data analysis pipeline, and the quality of the reference genome sequence is undoubtedly a major factor affecting the results. Several issues with the reference genome quality have been raised over the recent years. First, it is important to mention that the commonly used reference genome sequence originates from the initial assembly obtained by the Human Genome Project [77, 78]. The assembly has been updated multiple times; however, almost 10 years have already passed since the most recent update (GRCh38 or hg38) was published in 2013. Its predecessor, GRCh37 (or hg19), was introduced in 2009. GRCh38 and hg19 represent two of the most commonly used human reference genome assemblies. Both have several commonly used versions which differ in the number of extra-chromosomal sequences, patches and alternative contigs (ALT, representing alternative sequences of specific chromosomal loci). For hg19, the b37 version provided by the BROAD Institute is frequently used; similarly, a standard version of hg38 has also been suggested by BROAD and is included into the GATK resource bundle (https://gatk.broadinstitute.org/hc/en-us/articles/360035890811-Resource-bundle).

Despite the fact that almost 10 years have passed since the introduction of the GRCh38 reference, the majority of laboratories that use NGS in clinical settings still use the previous version [79]. Delay with switching to GRCh38 in medical genetics is motivated by the fact that many commonly used resources with genetic variant information, such as 1000 Genomes [80], ESP6500 [81] or the Exome Aggregation Consortium (ExAC) [82], were initially constructed using the b37 reference. The 1000 Genomes project has presented a hg38-realigned version of the data in 2017 [83]. For ExAC, an hg38-based allele frequency information appeared only after the emergence of the Genome Aggregation Database (gnomAD) [84]. Importantly, for the gnomAD v2.1.1, the hg38-based dataset has been produced by lifting over the b37-based VCF, which produces slightly different results compared with the complete realignment and re-analysis of the raw data. However, native hg38-based frequencies are available in the more recent releases, gnomAD v3 [85] and v4. In general, switching to the hg38 reference genome assembly introduced several notable improvements, in particular, in light of variant calling [86, 87]. However, even this assembly has its problems stemming from the approaches used to generate it (reviewed in [88]).

In 2022, a breakthrough in the human genome assembly was made by Nurk *et al*. [89] who presented the first fully complete reference assembly of the human reference genome sequence, named Telomere-to-Telomere (T2T). It has already been demonstrated that this assembly improves the results of various analyses, including genetic variation [90]. Unlike previous versions of the human reference genome, T2T contains no gaps or extrachromosomal sequences. Compared with the GRCh38, the majority of the added sequence in T2T corresponds to segmental duplications and centromeres, enabling thorough analysis of these regions [91, 92]. Additionally, the copy number of at least several hundred protein-coding genes has been updated. Given these improvements, T2T can be expected to become a new standard reference in human genomics; however, one can expect that switching to a new assembly may take even more time than the previous b37-hg38 transition.

Besides the general completeness of the assembly, there are at least two major issues with the widely used reference genome builds: (i) presence of minor alleles, including known disease variants, in the sequence [93]; and (ii) poor representation of the alternative sequence variants, especially in the hypervariable regions such as the major histocompatibility (MHC) locus. Both issues have attracted significant attention in recent years.

For the former problem, several solutions have been proposed, including construction of a major allele reference (e.g. [94]) or specialized variant callers (e.g. [95]). These solutions, however, are not widely used due to their incompatibility with the standard variant calling pipelines. We proposed an alternative method to deal with the reference minor allele (RMA) problem—RMAHunter, a tool to identify possible RMA-related false positive and false negative calls in a VCF file [93]. This solution, however, does not alleviate the RMA-induced errors at earlier analysis stages, e.g. during the read alignment.

**Figure 2:**
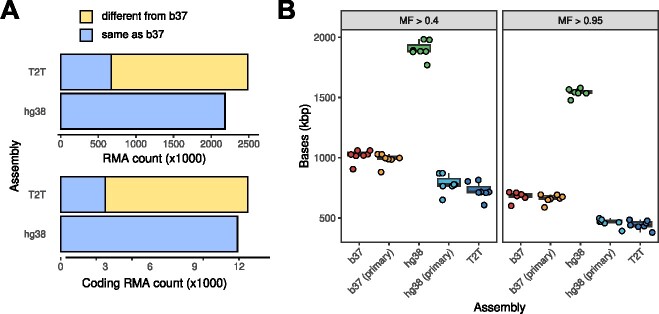
Presence of reference minor alleles (RMA) (A) and low-mappability regions (B) in the major human reference genome assemblies used for human genetic variation analysis—b37, hg38 (with either all or only primary contigs), and the new T2T assembly [89]. On (A), RMA sites in T2T are separated according to their concordance with hg38. On (B), the number of bases with a fraction of non-uniquely mapped reads > 0.4 (left) or 0.95 (right) is shown for the gold-standard GIAB WES datasets (HG001–HG007) used in our recent study [15].

As any individual genome contains a large number of singletons, i.e. rare variants that are uniquely present in an individual genotype [82, 84], one can expect a substantial number of RMA in any haploid genome assembly. Our earlier analysis identified as many as 2094 954 RMAs in the b37 reference, 12 709 of which were located inside the coding genome sequence. A follow-up analysis of RMA presence in hg38 and T2T shows similar results (Figure 2A), with 11 897 coding RMA sites in hg38 and as many as 12 587 such sites in T2T. Of note, only 3003 coding RMAs were shared between hg38 and T2T. An example of a potentially clinically relevant RMA site shared by all three assemblies is the rs587606297 frameshift variant in the *SCARF2* gene associated with the Van den Ende-Gupta syndrome (MIM 600920). The reference allele at this variant site has not been detected in any gnomAD individuals, making the presence of this allele in all three assemblies even more striking. In the whole genome, both hg38 and T2T contained above 2 million RMA sites, and, similarly, only 673 487 of these sites were shared (27.0%). These numbers illustrate the fact that RMA sites will remain in any consensus reference genome sequence; moreover, significant attention has to be paid when transferring the results to the T2T assembly given the substantial differences in nucleotide sequence.

While the RMA problem is inherent and almost unavoidable in any haploid consensus sequence, it may be solved by possible changes in the representation of the reference genome itself. One approach which is more and more commonly discussed is switching from a linear reference sequence to a graph-based genome representation. Such a reference genome graph should include most of the common sequence variants (and, especially, SVs), and is thus commonly referred to as a human pan-genome (reviewed in [96]). Alignment to a pan-genome graph is more computationally intensive compared with standard alignment to a linear reference; however, fast and accurate approaches to solve this task have recently been proposed (e.g. Giraffe [97] or the Seven Bridges GRAF pipeline [98]). An important advantage of the pan-genome-based approach is the ability to include all sequences that are relatively common in human populations but are missing from standard assemblies. Varying estimates of the proportion of such sequences have been made, though the majority of studies agree that each individual carries at least 0.5 Mbp of non-reference sequences [96]. The second benefit of the graph genome-based methods is their much higher accuracy in difficult-to-map MHC regions [99], as well as for the analysis of complex SVs. While complete switching to a pan-genome reference remains a remote perspective, increased application of long reads in medical genetics may also facilitate this process.

### Sequencing technologies and coverage biases

Another factor that affects the accuracy of variant discovery from NGS data is sequence coverage, i.e. the number of reads aligned to a particular position in the reference genome. Apart from the mean depth of coverage (the number of reads aligned to an average genomic position), it is also important to consider the breadth of coverage—the proportion of bases that are covered with sufficient amounts of reads to enable accurate variant analysis. Breadth of coverage is determined by the uniformity of coverage [100] and existing coverage biases, which are plentiful and differ between platforms [101].

The issue of coverage bias remains in focus of researchers since the emergence of NGS methods and, in particular, WES, with multiple comparative studies performed over the recent decade (e.g. [102]). Coverage is known to be significantly less uniform in WES compared with WGS, especially if WGS libraries are prepared with PCR-free methods [103]. GC-content is frequently considered as the leading factor that negatively affects the evenness of coverage in WES [102, 104, 105].

Our recent results confirm that coverage unevenness remains a significant issue in WES [101]. However, an in-depth analysis of the determinants of low coverage in WES and WGS shows that short read mappability limitations are the single most important determinant of CDS coverage in the human genome. Low-mappability regions span from 400 000 to over 1000 000 base pairs of the human coding sequence, and the mappability problem is more pronounced for WES compared with PCR-free WGS due to lower insert size in many WES libraries [101]. Exome kit design (i.e. inclusion or exclusion of particular regions from the intended target list) is also an important predictor of low coverage for WES [101].

The newest exome capture kits, such as Agilent SureSelect v8 or xGen Exome Panel, introduce improvements both in terms of design and general coverage uniformity [106, 107]. However, a substantial proportion of CDS bases remain non-targeted by the most recent WES kits (e.g. 1.4 Mbp for Agilent SureSelect v8 and 1.9 Mbp—for xGen Exome). Moreover, these non-targeted regions for both of the aforementioned platforms have very low overlap with low-mappability regions (e.g. only $\approx $ 11 000 bp overlap for Agilent SureSelect v8), indicating that the design of the WES baits remains a significant and independent factor affecting CDS coverage.

The aforementioned limitations of short read mappability are known to affect many important genes [101, 108]. Some of these, such as the spinal muscular atrophy genes *SMN1* and *SMN2*, are completely represented by low-mappability sequences, while other genes (e.g. neuromuscular disease-associated *NEB* or *TTN*) include multiple low-mappability parts. Indeed, the amount of bases with low mappability depends on the reference genome assembly used, with segmental duplications being one of the major sources of mappability issues. Importantly, assemblies also substantially differ in the span of regions completely covered by reads with multiple mapping locations (Figure 2B). When all hg38 contigs are used without the proper ALT-aware alignment, up to 1.5 Mbp of CDS sequences fall into the low-mappability category. This observation is consistent with earlier reports by other authors that emphasized the importance of ALT-aware alignment for comprehensive variant calling [60, 109]. When only primary contigs are considered, T2T consistently shows the minimal number of bases covered by non-unique mappers (median number is 430 000 bp), while b37 remains the worst with a median span of low-mappability regions of 662 000 bp. These figures further support the potential importance of the switching to a complete reference genome assembly in medical genomics. At the same time, it is important to note that no changes to human reference genome assembly can completely eliminate the mappability problem, emphasizing the importance of switching to long read sequencing technologies.

### Variant calling performance in challenging regions

State-of-the art variant callers demonstrate very good performance in recent benchmarks, with F1 scores well above 99% [15]. However, such a good result is likely explained by the fact that evaluation of the accuracy of variant calling is typically performed using high-confidence regions (reviewed in [99]). These sets of regions are maintained by GIAB for each of the seven gold standard samples (HG001-HG007), and represent the parts of the genome for which high-confidence ground truth variants are available. High-confidence regions broaden over time, owing to the GIAB continuous efforts to improve the dataset using various sequencing technologies, such as linked and long reads [110–112]. For example, high-confidence regions for the latest version of the GIAB dataset (v. 4.2, [112]) include 6% more bases compared with earlier versions, and the additional sequences correspond to various challenging regions. Still, the common (overlapping between all samples) high-confidence regions span only 2.37 Gbp (75.2% of the human genome sequence), and 30.4 Mbp of the coding sequence (86.6%) [15].

The remaining regions, spanning as much as 4.7 Mbp of CDS, are the most challenging, hard-to-call intervals. These regions include locations of SVs and segmental duplications, some of the low-mappability regions, as well as the major histocompatibility complex (MHC) locus [112]. Within the coding genome, the majority of such intervals reside on chromosome X, which is not included into the high-confidence intervals set for all samples except NA12878 (HG001). The X chromosome accounts for 1.3 out 4.7 Mbp of hard-to-call coding regions. Among the autosomes, the largest amount (0.4 Mbp) of hard-to-call regions are located on chromosome 19. Notably, only half of the low mappability regions are not included into the high-confidence set, which corroborates the description by Wagner *et al*. [112]. The set of genes corresponding to challenging autosomal regions overlaps CDS of 1116 genes. This list of genes is enriched with immune system and hemostasis genes, as well as signaling pathway components. Notable examples of medically important genes with challenging coding sequence include several members of the collagen gene family, such as *COL6A2* (linked to Ulrich myodystrophy (MIM 254090) and Bethlem myopathy (MIM 158810)), *COL27A1* (causal gene for the Steel syndrome, MIM 615155) and *COL11A2* (associated with deafness and other diseases of the ear).

How well do variant callers perform outside of the high-confidence regions? While the ground truth variants are not known for these regions, it is possible to evaluate the performance on challenging regions that are already included in GIAB v.4.2. Such an analysis performed by Olson *et al*. showed that most pipelines that were used in the precisionFDA Truth Challenge had a substantial drop in performance in difficult-to-map regions (up to 10% in F1 score), and the performance was only slightly better in the MHC region [113]. Similarly, a significant drop in performance can be seen when comparing the performance of the same pipelines on a broader (GIAB v. 4.2) and smaller (GIAB v. 3.3) set of high-confidence regions [15]. However, none of the aforementioned comparisons can provide insights into the performance of variant callers outside of GIAB v. 4.2 high-confidence regions.

**Figure 3:**
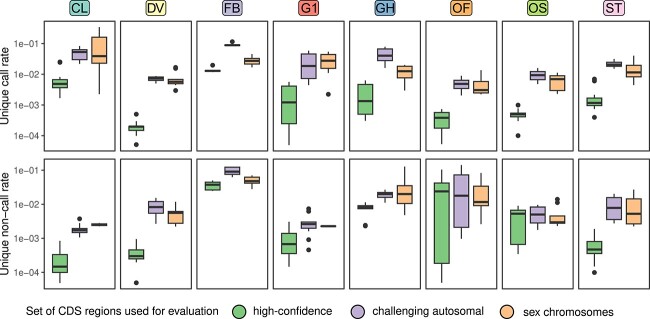
The performance of variant callers outside of the high-confidence benchmarking regions evaluated using the proportion of uniquely called (top) or uniquely non-called (bottom) variants (see [15] for the description of the metrics). The evaluation was performed using the gold-standard GIAB WES and WGS datasets (HG001–HG007). CL–Clair3, DV–DeepVariant, FB–Freebayes, G1 and GH–GATK HaplotypeCaller with 1D CNN or hard filtering, OF and OS–Octopus with either random forest or standard filtering, ST–Strelka2.

In our recent study, we proposed a truth set-free procedure to estimate the accuracy of variant calling by different tools using a set of concordance-based metrics [15]. For example, we showed that the number of variants uniquely called or uniquely missed by a particular variant caller is proportional to its general performance in a benchmark. Notably, our follow-up analysis shows that the percentage of unique calls and unique non-calls is up to 100 times greater outside of high-confidence regions (Figure 3). Furthermore, the results show that the proportion of uniquely called or uniquely missed variants is similar for challenging autosomal CDS regions and for sex chromosomes. Most callers report from 1 to 10% unique variants in non-high-confidence regions, although best-performing solutions (e.g. DeepVariant) tend to have a lower burden of unique calls and unique non-calls in all cases. These results support the findings of [112, 113], and suggest that variant calls outside of high-confidence regions have to be treated cautiously when analyzing and interpreting clinical NGS data. It is important to note that there are more than 4000 such variants within an individual exome (estimated using the HG001 GIAB dataset), and as many as 17 607 (12.9%) known pathogenic variants fall outside of high-confidence regions. These figures are greater than expected given the total length of these intervals, which is likely influenced by the hypervariable and clinically significant MHC region.

Moreover, the aforementioned results indicate that variants on chromosomes X and Y are also challenging for variant calling pipelines. This result is important given the large number of medically relevant genes located on chromosomes X and Y (e.g. *DMD*, *SRY*). For example, 10 604 known pathogenic variants are located on these chromosomes in ClinVar (v. 2023-03-26) (7.8% of all pathogenic variants, which is more than expected by chance (4.0%)). These numbers indicate that more attention has to be paid to variant calling and genotyping within sex chromosomes.

## BIOINFORMATIC METHODS FOR VARIANT ANNOTATION AND INTERPRETATION

Interpretation of the results is arguably the most challenging step in NGS-based molecular diagnostics of rare disease. The main goal of variant interpretation is to identify a single variant (or several variants) which are causal for the patient’s phenotype. This task is complicated by an enormous number of genetic variants identified in each single sample. Typically, three to four million variants are identified in an individual WGS sample, with around 25 000 variants found in CDS for both WGS and WES datasets [101]. The number is reproducibly higher for individuals of African ancestry (more than 30 000 CDS variants identified from a typical WGS experiment). Identification of a single causal mutation in such a large set of variants is not straightforward, and many tools have been proposed to assist with this process. Accuracy of variant interpretation greatly depends on both bioinformatic methods (e.g. computational annotation and prioritization of the identified variants) and clinical genetics factors (e.g. quality of the patient’s phenotype description, expertise of the person responsible for interpretation, or the genetic architecture of a particular disease). In this section, we will focus solely on bioinformatic aspects of efficient variant interpretation; however, it is important to emphasize the critical importance of a correct clinical diagnosis and phenotype description for the successful variant interpretation.

Multiple sets of guidelines and recommendations for interpretation of NGS results in medical genetics have been developed. American College of Medical Genetics and Genomics (ACMG) guidelines [114] are considered the gold standard for germline variant interpretation. These guidelines include a fixed number of criteria which assess the potential pathogenicity of a variant based on different kinds of supporting evidence. A set of rules is then applied to classify a variant into one of the five categories based on these criteria: pathogenic, likely pathogenic, likely benign, benign or variant of uncertain significance (VUS). Web-based platforms such as Franklin (https://franklin.genoox.com/clinical-db/home) have been developed to automatically classify a given variant according to ACMG criteria. Further extensions of the ACMG guidelines (e.g. Sherloc [115]) have been proposed to formalize the classification procedure based on numerical scoring schemes. However, fully automated classification of variants remains challenging.

### The basics of variant annotation: transcripts and allele frequencies

Correct interpretation of results relies on accurate and comprehensive annotation of genetic variants with the relevant information available. There are three most commonly used tools for variant annotation—SnpEff [116], ANNOVAR [117, 118] and Ensembl Variant Effect Predictor (VEP) [119]. Google Scholar Citations (accessed 2023/04/17) indicates that ANNOVAR is the most cited tool, with more than 11 000 citations to date.

It has previously been noted that the annotation software may yield different results for the same query variant [120, 121]. One reason for the discordance in annotation is the differences in the genome annotation (sets of transcripts) used during annotation. According to earlier reports, the discordance between annotations may amount to as much as 90% for certain classes of variants, and more than 40% for putative loss-of-function (pLoF) variants [120]. While the discordance rate between the tools is lower than between annotations, the concordance of annotations for indel variants is rather low (less than 90%) [121].

ACMG guidelines for variant interpretation suggest that the variants should be interpreted according to the National Center for Biotechnology Information (NCBI) RefSeq [122] or Locus Reference Genomic (LRG) [123] transcript sequences [114]. At the same time, global resources of genetic variation such as gnomAD or National Heart, Lung, and Blood Institute (NHLBI) TopMed [124] report variants according to Ensembl/GENCODE [125, 126] transcripts. Moreover, a richer set of Ensembl transcripts may be helpful in certain complex cases. For example, Schoch *et al*. described a pathogenic effect of the deletion in the *KMT2C* gene causing Kleefstra syndrome type 2 (MIM 617768) [127]. Other deletions in the same gene are present in healthy individuals; however, such deletions only affect proximal exons of the RefSeq transcript (NM_170606.3). Ensembl, however, lists additional shorter transcripts for the same gene (e.g. ENST00000424877.5 and ENST00000360104.7), which are not affected by common deletions but are disrupted by a pathogenic deletion in the patient.

In light of the aforementioned data, it is not surprising that different sets of transcripts are used both in non-clinical and clinical practice [79], indicating a need for a unified transcript annotation for clinical genetics. Finally, a joint effort was made in 2022 by NCBI and EMBL-EBI to construct such a set of manually curated transcripts for clinical genetics called Matched Annotation from NCBI and EMBL-EBI (MANE) [128]. Switching to this transcript set would greatly decrease the burden of genome annotation differences.

Besides the choice of transcripts for annotation, allele frequency in the population is also important for ascertaining the pathogenicity of a variant. Allele frequency information helps to filter out many candidate variants which are actually benign and present at a high frequency in healthy individuals. The allele frequency information is usually obtained from large-scale global sequencing projects which provide publicly available summary data, such as the 1000 Genomes project [80], or gnomAD [84]. While global frequency information is plentiful, local allele frequency information is also desirable. It has previously been shown that using the maximum allele frequency across populations (popmax) helps to limit the set of candidate variants for interpretation [82]. Furthermore, our analysis demonstrated the utility of population-specific variants for identification of low-confidence pLoF variants [129]. Multiple ancestry-specific allele frequency databases have been established worldwide (e.g. [130–132]). A substantial number of variants absent from global population databases can be present in healthy donors from underrepresented populations [131], emphasizing the importance of ancestry-specific information for accurate annotation and prioritization of variants.

### Multi-nucleotide polymorphisms

Multi-nucleotide polymorphisms (MNP) are combinations of single-nucleotide substitutions in two or more adjacent positions. If several substitutions that constitute an MNP are located within the same codon, their effect on the coding sequence has to be analyzed jointly. However, a default behavior of variant annotation tools is to consider each of the called genetic variants separately, thus leading to potential misinterpretation of the effects of MNPs. Despite the generally low density of genetic variants in an individual genome, several dozen MNPs are present in any sample as shown by ExAC and gnomAD [82, 133]. The problem of MNPs becomes even more important if RMA are taken into account. As shown in our analysis [93], more than 10 000 coding variants can be misclassified due to the presence of an RMA in the same codon. This number also includes known pathogenic variants, such as the rs104894197 variant in the *ALX4* gene for which the expected consequence changes from nonsense (p.Ser270*) to missense (p.Ser270Trp) if a neighboring synonymous RMA site (rs10769028) is accounted for. Specialized tools for annotation of MNP effects have been developed (e.g. COPE [134]), but have not become widely used. Confident identification of MNPs also requires genotype phasing, as two heterozygous SNVs in the same codon might be located on different haplotypes [133].

### Challenges in predicting the loss-of-function potential of coding variants

Loss-of-function (LoF) is the main mechanism of rare disease pathogenesis. Hence, identification of protein-coding genetic variants with LoF effects is crucial for diagnostics of rare disease. Three classes of genetic variants are usually considered as pLoF variants: nonsense (stop_gained) variants, frameshift insertions and deletions and canonical splice site variants. These variants are also called protein-truncating variants (PTVs), as most of such variants lead to premature termination of protein synthesis. Despite the anticipated large effects of PTVs on gene function, each individual carries up to 100 such variants [82], indicating that additional filters are required to identify genuine LoF variants among the PTVs. Several rules for filtering out low-confidence LoF (LC LoF) variants have been developed, and variant annotator plugins such as LOFTEE [84] have been implemented to classify pLoF variants according to these rules. For example, LOFTEE considers variants within the 5% of the end of the coding sequence as LC LoF. Similarly, an LC LoF flag is assigned to variants in canonical splice sites for introns less than 15 bp long.

However, predefined sets of rules are insufficient to filter out all false positive LoF variants. Gene expression data are also widely used to determine the actual LoF potential of a variant. A recently proposed proportion of expressed transcripts (pext) score [135] utilizes gene expression information from the Genotype Tissue Expression [136] to identify and exclude variants that only affect isoforms with low or zero expression levels. However, as shown in our recent analysis of population-specific PTVs [129], pext is not sufficient to filter out all LC LoF variants. Other approaches for classifying pLoF variants have also been proposed. One example is a machine-learning-based algorithm called MutPred-LoF [137], which showed high efficiency in predicting pathogenicity of nonsense and frameshift variants. Nevertheless, the predictive power of the proposed methods is still far from ideal.

The problem of predicting the LoF effects is significantly more complex for missense variants. Several types of evidence are usually used to solve this task, including data on evolutionary conservation of protein sequences, 3D protein structure and chemical properties of the reference and mutant amino acids. Dozens of bioinformatic tools for prediction of pathogenicity of missense variants have been proposed. The most commonly used algorithms (according to Google Scholar Citations) are SIFT [138, 139], PolyPhen2 [140] and CADD [141]. The large number of tools and high levels of inconsistency, however, led to the emergence of algorithms that aggregate predictions from different software (e.g. REVEL [142] or [143]). Moreover, databases that store pre-computed predictions have been constructed, such as dbNSFP [144]. New algorithms, however, are still being actively developed (e.g. MutPred2, [145]), and strategies for interpreting pathogenicity prediction scores in the ACMG framework have been proposed [146].

Besides missense and other high-impact variants in the coding sequence, other variants that are usually considered silent (i.e. not affecting the gene product function) can also lead to loss of protein function. Multiple examples of unexpected functional effects of synonymous variants have been reported, and different mechanisms of pathogenic effects of these variants have been described. These include introduction or disruption of splice sites and negative effects on translation rate (e.g. [147]), For example, a synonymous variant NM_001077488.2:c.108C>A [p.Val36Val] in the *GNAS* gene has been reported to result in a cryptic splice site leading to missplicing of the corresponding transcript and causing pseudohypoparathyroidism [148]. Development of computational predictors for synonymous variants is complicated, though several efforts have been made in this direction (e.g. syn-Vep [149]).

One of the important factors that may affect the performance of predictive algorithms is the specificity in the intragenic localization of pathogenic variants. Multiple examples of clustering of pathogenic variants in specific gene regions have been noted. For example, pathogenic variants that cause Floating-Harbor syndrome are uniquely localized in the 34th exon of the *SRCAP* gene, as confirmed in recent reports [150, 151]. The issue of non-uniform distribution of pathogenic variants has attracted attention of researchers for many years (e.g. [152]). Recently, Laddach *et al*. investigated the specific patterns of the distribution of pathogenic missense variants compared with neutral ones [153]. In particular, Laddach *et al*. found that pathogenic variants are enriched in protein cores and protein interaction sites, in contrast to both common and rare variants from a healthy population. However, further research is required to create a comprehensive map of clinical significance for each site in disease genes.

### Annotation and interpretation of noncoding genetic variants

**Figure 4:**
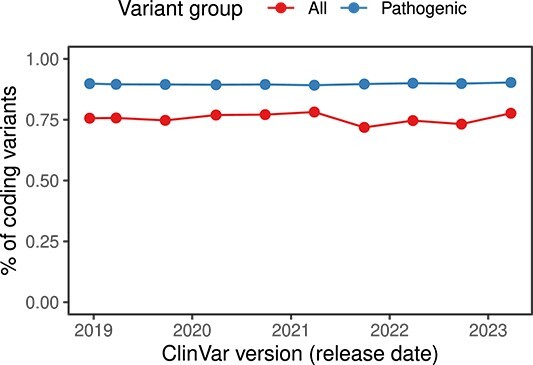
The proportion of coding variants among the pathogenic ones in the ClinVar database remains constant over the last 5 years. The numbers were estimated using the corresponding weekly ClinVar versions and the GENCODE v43 human genome annotation.

It is often hypothesized that one of the reasons explaining the low diagnosis rate of inherited disease from NGS is the inability to interpret the pathogenicity of variants located outside of the coding genome sequences [4]. Indeed, the noncoding variants are dramatically underrepresented among high-confidence pathogenic variants across all databases [154]. Moreover, our analysis shows that the proportion of noncoding variants among the known pathogenic ones remains constant over the last 5 years (Figure 4), likely reflecting both a limited proportion of noncoding variants among the true disease-causing ones as well as the lack of evidence to classify noncoding variants as pathogenic.

In contrast to protein-coding variants discussed in the previous section, computational prediction of the functional consequences of noncoding variants is even more challenging. However, multiple methods have recently emerged to make such predictions. These methods are based on two principal types of evidence: (i) the level of evolutionary conservation and sequence context of the corresponding genomic region; and (ii) effect of variant on known features of the noncoding genome.

In the first category, generic models for the analysis of evolutionary conservation of genomic regions, such as GERP++ [155] or phastCons [156], can be leveraged for prioritization of noncoding variants. Furthermore, specialized predictors of deleterious effects of noncoding variants based on sequence context and evolutionary conservation status have been proposed (e.g. FATHMM-MKL [157]). Among the functional element-specific methods, much of the attention is focused on evaluating the functional effects of intronic variants, variants affecting known regulatory elements, variants in the 5’- or 3’-untranslated regions (UTRs), and variants affecting annotated noncoding RNA sequences. For intronic variants, SpliceAI has become a widely used standard tool for predicting the effects of variants on splicing [158]. For UTR variants, tools to predict the impact of such variants on the function of upstream open reading frames (uORFs) have been developed [159, 160].

Despite the fact that interpretation of the noncoding variants remains a significant problem for over a decade, the first ACMG-based guidelines for noncoding variant interpretation were published only in 2022 [154]. Notably, these guidelines suggest that only known elements of the noncoding genome (UTRs, introns, promoters and other cis-regulatory elements) functionally linked to the target gene have to be considered during interpretation to decrease the amount of candidate variants. Moreover, evidence supporting the role of a particular element of the noncoding genome should be present in relevant tissues and cell types. These rules predicate a need for the development of new context-aware tools for annotation of noncoding variants. Such tools should consider the functional relevance of the genomic element of interest as well as the impact of a particular variant on this element.

### Periodic reanalysis of data for undiagnosed cases

It is also important to emphasize the necessity of periodic re-analysis of the data for undiagnosed cases, as suggested by multiple studies (e.g. [161, 162]). The increase in diagnostic rates from reanalysis varies but is usually substantial. A literature review by Tan *et al*. showed a median rate of new diagnoses of 15% [163]. Some studies also report improved clinical outcomes for patients who received a diagnosis upon reanalysis [164]. Given the pace of new technological advancements, reanalysis of undiagnosed cases can become even more effective. However, we hope that new sequencing technologies and bioinformatic approaches will help to significantly decrease the number of undiagnosed cases after genomic diagnostics.

## CONCLUSION

As demonstrated in this review, NGS data analysis and interpretation remains a daunting task, and various factors negatively affect the overall diagnosis rate for NGS-based rare disease diagnostics. Among these, variant calling in challenging genome regions, defined by limitations of short read sequencing, appears as the most influential factor, affecting up to 15% of all coding sequences in the human genome and more than 12% of all known pathogenic variants. To reduce the impact of this issue on routine data analysis and variant interpretation, one might be advised to use the best-performing combination of software (e.g. BWA MEM aligner and DeepVaraint caller) and be more cautious when interpreting variants in challenging regions. For example, the reliability of the corresponding region should be considered during interpretation (e.g. using a scoring system such as DangerTrack [165]). While other factors, such as errors in the reference genome or annotation inconsistency, seem less influential, these issues also have to be borne in mind upon interpretation to avoid costly mistakes.

As discussed above, a substantial fraction of undiagnosed cases may be due to a lack of biological knowledge of rare disease mechanisms. It seems unlikely that this problem can be solved in the nearest future, though large-scale genomic projects focused on rare disease genetics are of great importance in this context. For example, numerous new findings have been made by the 100 000 Genomes Project by Genomics England (e.g. [166, 167]). Further research into the genetic causes of rare disease may be expected to significantly increase the diagnosis rates for NGS-based diagnostics. In addition to the identification of new disease genes, functional genomic studies into the pathogenetic mechanisms of inherited disorders are also of crucial importance. In particular, investigation of the epistatic interactions between genes, as well as complex gene-environment interactions, may be helpful to predict the functional and clinical effects of genetic variants in individual genomes.

Besides the lack of biological knowledge, diagnosis rates are influenced by technical limitations of the short read-based NGS technologies and linear reference-based analysis methods. The ongoing process of making long read sequencing available for clinical diagnostics can be expected to solve many of the problems mentioned in this review (discussed in more details in [99, 168]). If these developments are combined with the recent breakthroughs in the reference genome assembly, as well as efforts to construct a human pan-genome [169, 170], comprehensive analysis of all genetic variants present in a particular individual may be achieved in the upcoming years.

**Key Points**

- Identification of germline genetic variants from NGS data involves several stages: read alignment, alignment preprocessing and variant calling. Each of these steps could be performed with various tools; BWA and DeepVariant appear to be the most accurate aligner and variant caller.
- Inaccurate variant calling in challenging genomic regions is the most influential factor that negatively impacts variant discovery in as much as 4.7 Mbp of the coding genome sequences. Mappability limitations of short reads and reference minor alleles also affect the results, but to a lesser extent.
- Prediction of the deleterious effects of genetic variants, both inside and, especially, outside of the coding genome, is one of the most important challenges in bioinformatic variant annotation. Other important issues in this area include the completeness and consistency of the genome annotation and the availability of local allele frequency information.
- Further research into the molecular mechanisms of inherited disease combined with wider adoption of long read sequencing and a switch to the complete human genome assembly (or the human pangenome reference) will help to overcome the major persistent challenges.

## Author Biographies

**Yury Barbitoff** is the bioinformatics group leader at the Dpt. of Genomic Medicine of the D.O.Ott Research Institute of Obstetrics, Gynaecology, and Reproductology since 2019, and served as a director of research at the Bioinformatics Institute from 2017 to 2022. The main field of research of Dr. Barbitoff’s group is the development of new tools for the analysis of next-generation sequencing data in human genetics. The group has a specific focus on the development and application of bioinformatic methods for rare disease diagnostics.

**Mikhail Ushakov** is a staff scientist at the Dpt. of Genomic Medicine of the D.O.Ott Research Institute of Obstetrics, Gynaecology, and Reproductology. His work is mainly related to the development of bioinformatic pipelines for variant calling from NGS data.

**Tatyana Lazareva** works as a staff scientist at the Dpt. of Genomic Medicine of the D.O.Ott Research Institute of Obstetrics, Gynaecology, and Reproductology. Her research focus is the analysis of various factors that determine the impact of genetic variants on the phenotype. She also develops new methods for NGS data analysis and interpretation in medicalgenetics.

**Yulia Nasykhova** is the head of the Genomics laboratory at the Dpt. of Genomic Medicine of the D.O.Ott Research Institute of Obstetrics, Gynaecology, and Reproductology. Dr. Nasykhova’s laboratory conducts research in the field of human genetics and maintains the “Human Reproductive Health” bioresource collection at the institute.

**Andrey Glotov** is the Head of the Dpt. of Genomic Medicine of the D.O.Ott Research Institute of Obstetrics, Gynaecology, and Reproductology. Dr. Glotov supervises all research at the Department, which is dedicated to various aspects of humangenetics, with a specific focus on the genetic aspects of human reproduction.

**Alexander Predeus** is currently a principal bioinformatician at the Wellcome Sanger Institute, UK. His current work is focused on developing novel methods for the analysis of single cell data. During his tenure with Bioinformatics Institute (2015-2017) Alexander served as director of science and worked on human genomics and its applications to rare disease. Alexander is currently a scientific advisor at Bioinformatics Institute.
